# Low genetic diversity and predation threaten a rediscovered marine sponge

**DOI:** 10.1038/s41598-022-26970-w

**Published:** 2022-12-28

**Authors:** Z. B. Randolph Quek, Juat Ying Ng, Sudhanshi S. Jain, J. X. Sean Long, Swee Cheng Lim, Karenne Tun, Danwei Huang

**Affiliations:** 1 Department of Biological Sciences, National University of Singapore, Singapore, Singapore; 2 Yale-NUS College, National University of Singapore, Singapore, Singapore; 3 School of Design and Environment, National University of Singapore, Singapore, Singapore; 4 National Biodiversity Centre, National Parks Board, Singapore, Singapore; 5 Republic Polytechnic, Singapore, Singapore; 6 Tropical Marine Science Institute, National University of Singapore, Singapore, Singapore; 7 Lee Kong Chian Natural History Museum, National University of Singapore, Singapore, Singapore; 8 Centre for Nature-Based Climate Solutions, National University of Singapore, Singapore, Singapore

**Keywords:** Biodiversity, Conservation biology

## Abstract

Discovered in 1819 in the tropical waters off Singapore, the magnificent Neptune’s cup sponge *Cliona patera* (Hardwicke, 1820) was harvested for museums and collectors until it was presumed extinct worldwide for over a century since 1907. Recently in 2011, seven living individuals were rediscovered in Singapore with six relocated to a marine protected area in an effort to better monitor and protect the population, as well as to enhance external fertilisation success. To determine genetic diversity within the population, we sequenced the complete mitochondrial genomes and nuclear ribosomal DNA of these six individuals and found extremely limited variability in their genes. The low genetic diversity of this rediscovered population is confirmed by comparisons with close relatives of *C. patera* and could compromise the population’s ability to recover from environmental and anthropogenic pressures associated with the highly urbanised coastlines of Singapore. This lack of resilience is compounded by severe predation which has been shrinking sponge sizes by up to 5.6% every month. Recovery of this highly endangered population may require ex situ approaches and crossbreeding with other populations, which are also rare.

## Introduction

The marine fauna of the world is facing an extinction crisis^1–3^, with various anthropogenic pressures such as climate change and overharvesting driving losses in animal species living in the sea^4–6^. Compounding the problem, modern threats such as coastal urbanisation are wiping out entire populations of marine animals^7,8^. On the conservation front, well-studied fauna such as corals receive much of the limelight, typically with ominous predictions^9,10^. Nevertheless, a comprehensive review on sponges found no pressing need for special provisions to be made for most species globally, albeit acknowledging the lack of information on anthropogenic effects could be to their detriment^11^. Furthermore, recent studies predict sponges will outlast the onslaught of warming oceans wrought by climate change, even with impacts such as anoxic conditions caused by eutrophication^4,12,13^. Conversely, opinions on the vulnerability of populations in the near future have been gaining traction^14–20^.

**Figure 1:**
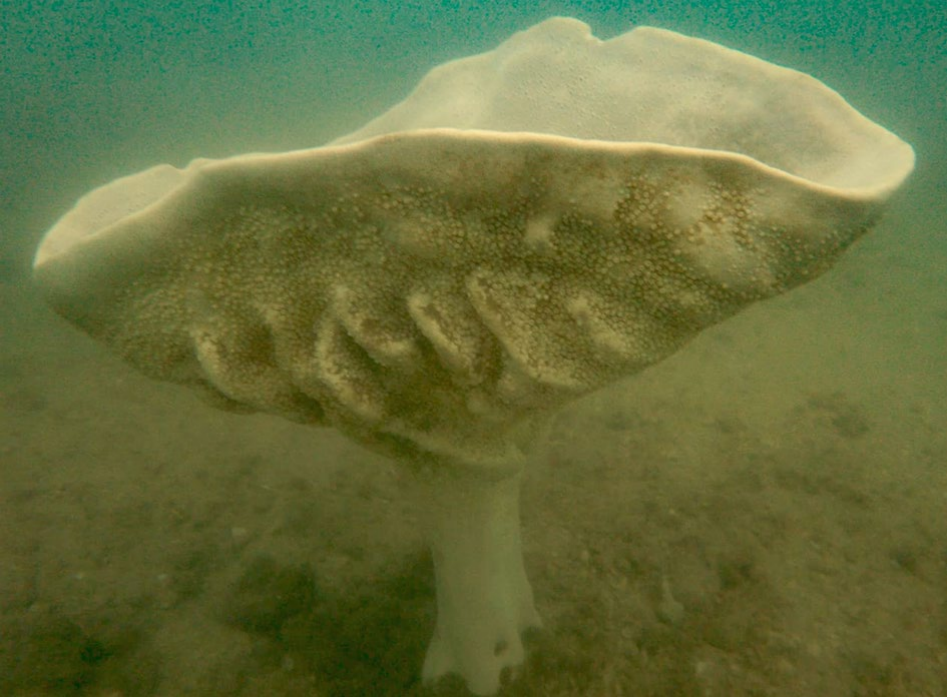
A live specimen of *Cliona patera* in Singapore, photographed in November 2018.

With over 9000 species found throughout the world’s marine ecosystems^21^, sponges form integral components of benthic environments. They not only stabilise coral reefs via bioerosion and substrate consolidation, but are also heavily involved in nutrient cycling^22,23^. Recent reviews on the ecology of sponges emphasised their critical contributions to carbon, nitrogen and phosphorous biogeochemical cycling^24,25^. Furthermore, sponges are a rich source of secondary metabolites, with potential for novel compounds as drug candidates^26–29^. Despite their ecological and economic importance, many populations along coastlines are facing declines, having to endure a barrage of problems such as urbanisation, pollution, and overfishing^20,30–32^. Exemplifying the negative effects of anthropogenic-related pressures on natural populations in Southeast Asia, the magnificent Neptune’s cup sponge *Cliona patera* (Hardwicke, 1820) was harvested for museums and collectors until it became presumably extinct with the last living specimen recorded in 1907 off the coast of present day Banten, Indonesia^33^. Notably, in the nineteenth century, *C. patera* was recorded to be present in large numbers in Singapore, whose native people used to collect the sponges in droves for Europeans demanding specimens due to the species’ unique morphology^34^ (Fig. 1).

Since the 1990s, specimens of *C. patera* from Australia and Southeast Asia have emerged from trawling and biodiversity surveys, suggesting that at least some remnant populations persisted^26,34–37^. Indeed, *C. patera* was rediscovered in 2011 from its type locality, Singapore, with just seven living individuals that are currently known to exist. Coastal populations of marine fauna are more adversely impacted by human activities^31,32^, which could reduce the rate of population recovery of *C. patera* in Singapore. In a bid to conserve this species, six of the seven sponges have been relocated to the Sisters’ Islands Marine Park (SIMP), a marine protected area managed by Singapore’s National Parks Board. Within that area, strict protection laws are enforced, and the site facilitates regular monitoring efforts. There is an added benefit of the aggregation which enhances the probability of external fertilisation in benthic organisms^38–41^. However, aggregating the sponges in a protected area might result in increased predation, particularly from large predators such as turtles, thus adversely hampering sponge recovery. Furthermore, small populations generally suffer from low genetic diversity^42^, limiting the long-term viability of this sponge population. The efficacy of this measure in recovering the local population of this critically endangered species has hitherto not been assessed^43^.

This study aims to determine the genetic diversity of the local *C. patera* population across multiple loci, thereby estimating their resilience. We further characterised its ecology at the relocated site to assess risks and threats to the threatened population. With some individuals facing severe predatory stress on relocation, exacerbated by negligible population genetic variability, we propose urgent recovery and conservation measures for this enigmatic species to prevent it from becoming extinct, again.

## Methods

### Sampling and DNA sequencing

Tissue samples of 2 cm^3^ were taken from six relocated *C. patera* individuals and fixed in 100% molecular-grade ethanol. Genomic DNA and library preparation were conducted following Quek et al*.*^44^. Briefly, genomic DNA (gDNA) was extracted using EZNA Mollusc DNA Kit with a modified elution protocol to ensure high quality gDNA was obtained. Following which, purification of gDNA was conducted using Zymo DNA Clean-up and Concentrator Kit. Bioruptor Pico (Diagenode) and KAPA dual-indexed adapters were ligated to sheared fragments using KAPA HyperPrep kit. Final libraries were size-selected following manufacturers’ protocol using Agencourt AMPure XP beads (Beckman Coulter). The samples were then pooled in equimolar concentrations with other libraries not associated with this study and sequenced on a single Illumina HiSeq 4000 (150 × 150 bp) lane to recover the complete mitochondrial genome and nuclear rRNA sequences.

Separately, amplification of the internal transcribed spacer (ITS) for sponges was conducted using ITSRA2 (5′-GTC CCT GCC CTT TGT ACA CA-3′) and ITS2.2 (5′-CCT GGT TAG TTT CTT TTC CTC CGC-3′)^45^. Amplified products were purified using AMPure XP (Beckman Coulter) and cycle sequenced in both directions separately using the BigDye™ terminator method and sequenced using the ABI 3130 XL Genetic Analyzer (Applied Biosystems). Chromatograms produced were then edited on Geneious Prime (Biomatters) and assembled de novo.

### Genome skimming and loci identification

**Figure 2:**
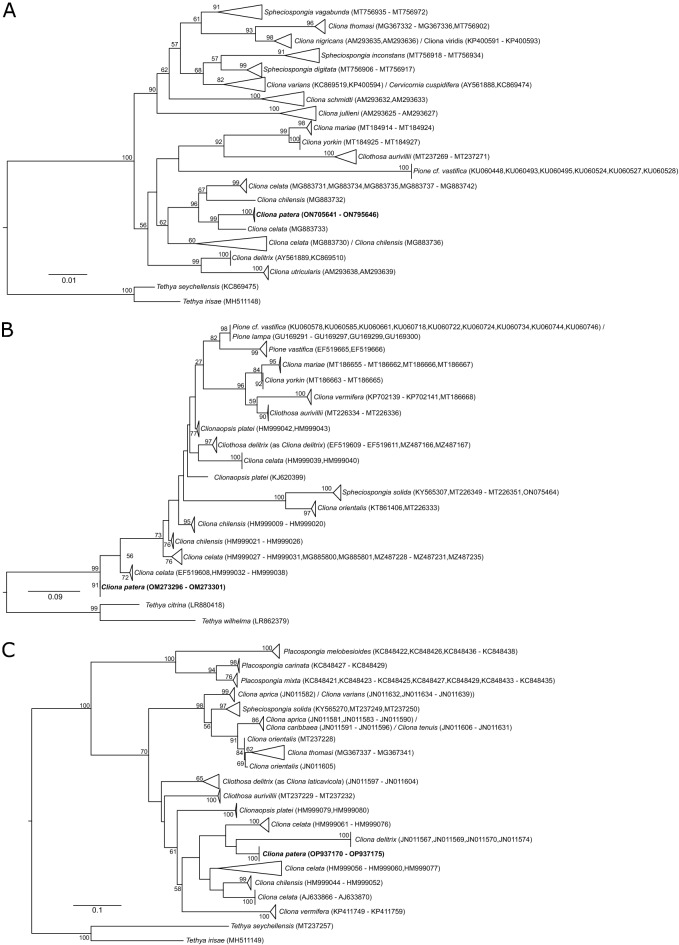
Maximum likelihood phylogeny of Clionaidae, with *Tethya* spp. as outgroup, based on three genes: (**A**) nuclear 28S rRNA; (**B**) mitochondrial cytochrome c oxidase subunit I (COI); (**C**) nuclear internal transcribed spacers (ITS). Numbers on nodes represent bootstrap support (≥ 50 only). Samples in bold represent those sequenced in this study.

Raw reads were demultiplexed and both low quality bases and adapters were trimmed and assembled using fastp v0.20.1^46^ and SPAdes v3.12.0^47^ respectively, under default settings. Mitochondrial genomes were identified by BLASTn (e-value 10^−6^), searching against other clionaid cytochrome c oxidase subunit I (COI) sequences downloaded from GenBank (Fig. 2), with the longest contig selected as the mitochondrial genome, and circularisation of the genome was executed by *circules.py*^48^. Annotation of mitochondrial genomes was conducted using MITOS2^49^.

Sequences of nuclear ribosomal gene 28S were identified in a similar fashion to that of COI, by searching against poriferan 28S sequences downloaded from GenBank using BLASTn (e-value 10^−6^) (Fig. 2). Only contigs with ≥ 200 bp overlap of ≥ 90% similarity to the references and minimum *kmer* coverage of 100 (determined by SPAdes v3.12.0) were extracted and separately assembled in Geneious Prime (Biomatters). Finally, to ensure overlapping regions between the sequences downloaded from GenBank with the assembled ribosomal sequences, we repeated the BLASTn search and extracted the overlapping region with the highest bitscore from the contig.

### Pairwise sequence comparisons

To compute pairwise distances among members of Clionaidae according to the World Porifera Database^21^, sequences of the following genes—COI, ITS, and 28S rRNA—were downloaded from GenBank (Fig. 2). To account for inconsistent identification of sponges that could inflate intraspecific distances, we first reconstructed a maximum likelihood (ML) phylogeny for each gene separately with *Tethya* spp. as outgroup. Sequences downloaded were aligned by MAFFT v7.271 under *–auto *settings^50^. Alignments were manually inspected to check for overlap between individuals of each nominal taxon. The best model of DNA evolution was identified using ModelTest-NG^51^ and specified in ML phylogeny reconstruction using RAxML-NG v0.8.1 with 10 random and 10 parsimonious trees, and 200 bootstrap pseudoreplicates^52^. Each gene tree was inspected, and we only kept sequences from named taxa that were recovered as a monophyly with a minimum bootstrap support value of 50 for downstream pairwise comparisons. Retained sequences were realigned by MAFFT as above and the alignments were imported into MEGA X^53^ to compute intraspecific pairwise distances.

### Ecological data

#### Growth

Monthly photographs of relocated sponges were tracked each month from June 2020 to December 2020 to estimate growth rates via SCUBA dive surveys. Photos were taken with a scale from a fixed distance and angle using an Olympus TG-5. The total height of each sponge was measured using ImageJ^54^. For months where visibility in the photo was low, the data were not used. Growth rate (cm/month) was computed based on the height data obtained monthly.

#### Regeneration capability

To determine the regeneration ability of *C. patera*, we cored the cup section of all six relocated individuals using a 2 cm diameter stainless steel apple corer and estimated the rate of tissue recovery in relocated *C. patera* individuals. Weekly surveys were performed until the hole had fully recovered. During each survey, the diameter of the cored hole was measured, and any other visual observations noted.

The surface area of the remaining hole was calculated based on the measured diameter. Recovery rate was measured by taking the difference in surface area every week, normalised by the number of days between two surveys. Average recovery rate of each sponge was calculated until the holes had completely sealed during the survey.

## Results

### Mitochondrial genome assembly and genetic diversity

**Figure 3:**
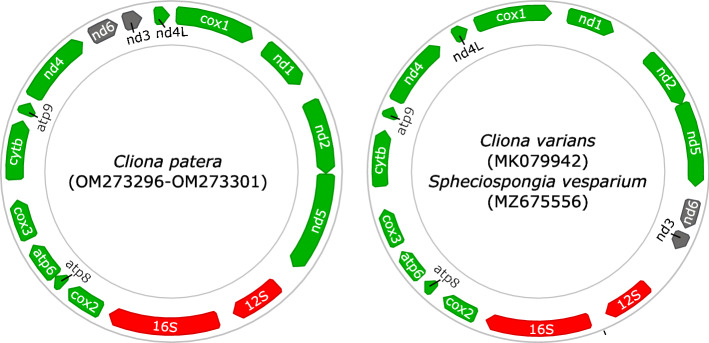
Circular maps depicting the mitochondrial gene arrangement in Clionaidae, partially reconstructed using Geneious Prime (Biomatters). Rearrangements between *Cliona patera* and confamilials are highlighted in grey. Annotation of *C. varians* and *Spheciospongia vesparium* was conducted using MITOS2^49^ as GenBank records were not formally annotated.

The mitochondrial genomes of all six samples sequenced were identical, with a length of 19,133 bp. Comparison of gene order of ribosomal and protein-coding genes (PCGs) between *C. patera* and congener *C. varians* found slight differences, with the block of genes comprising nad6 and nad3 adjacent to the 12S rRNA gene in *C. varians*, whereas it is between nad4 and nad4L in *C. patera* (Fig. 3).

**Figure 4:**
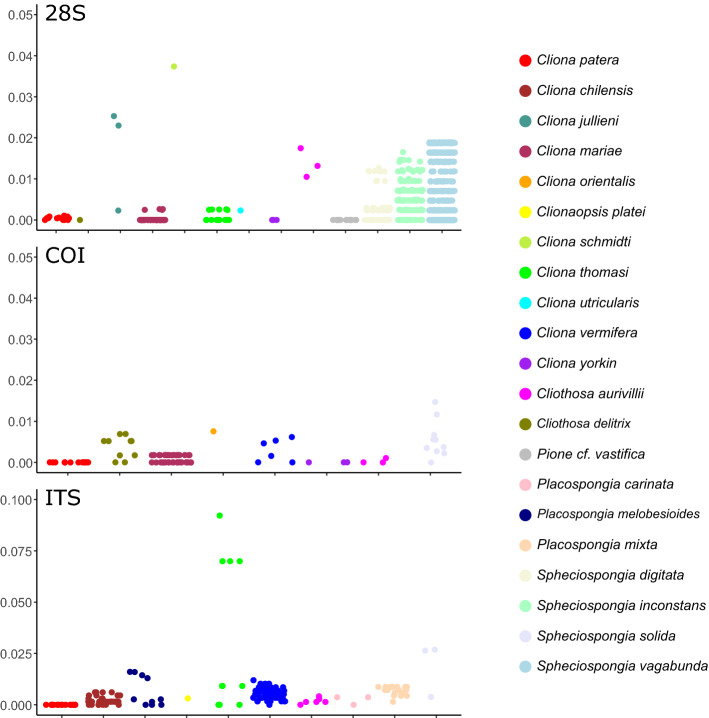
Intraspecific pairwise distances of clionaid species for three genes: nuclear 28S rRNA (top); mitochondrial cytochrome c oxidase subunit I (COI; middle), and nuclear internal transcribed spacers (ITS; bottom).

The phylogeny reconstructed showed a number of inconsistencies in taxon identification based on GenBank data (Fig. 3), corroborating studies such as those identifying cryptic species complexes in *C. celata*^55,56^. After extracting and analysing sequences only from taxa that form monophyletic groups, we found higher intraspecific pairwise differences in other clionaids, compared to genetic distances from nuclear and mitochondrial loci of *C. patera* that were nearly zero (Fig. 4, Tables S1–S3). Specifically, mean intraspecific pairwise distances for 28S rRNA locus in *C. patera* stood at 0.0423% (± SD 0.0324%), and no variability was detected for ITS and COI sequences.

### Growth and recovery

**Figure 5:**
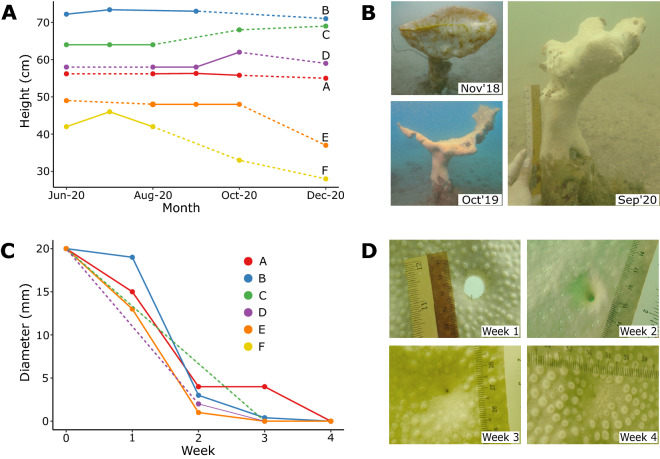
(**A**) Changes in height of *Cliona patera* sponges A–F over six months; (**B**) images showing changes to sponge F; (**C**) changes in diameter of 20 mm bored hole in sponges A–E over four weeks; sponge F was omitted due to consumption of bored area after just two weeks; (**D**) sponge B demonstrating full recovery by the fourth week. Solid and dotted lines connect between points with and without monthly data respectively.

Of the six sponges observed over a period of six months, three of them (A, B, D) maintained relatively constant heights (Fig. 5A) and one individual (C) grew appreciably by 5 cm (8%). For the remaining two individuals (E and F), we recorded a loss of between 12 and 14 cm (24–33%), likely due to severe predation. Their cups were almost entirely consumed and visible bite marks were recorded on their stems (Fig. 5A,B). Nevertheless, *C. patera* generally exhibited remarkable rates of recovery after boring, sealing the 20 mm diameter core hole within three weeks (Fig. 5C,D). However, one of the individuals (F) did not show any sign of recovery after two weeks, and by the third week of observation, the tissue at its core had been consumed by predators.

## Discussion

This study has provided clear evidence for two main threats against the long-term viability of the *C. patera* population in Singapore: low genetic variability and heavy predation pressure. Predation was so severe in some individuals that the entire cup was consumed, and would likely take a long time to recover, if at all possible. Indeed, sponge E is no longer alive (died sometime in February 2022) and only a small area of living tissue remains for sponge F. The known population in Singapore now stands at just six individuals.

The mitochondrial genome of *C. patera* presented a novel gene rearrangement when compared to confamilials *C. varians* and *Spheciospongia vesparium*, with the block nad6-nad3 being translocated to between nad4 and nad4l in the former, and being between nad5 and 12S rRNA in *C. varians*. Examination of mitochondrial gene arrangements in Demospongiae has revealed multiple rearrangements, and is particularly rife in Heteroscleromorpha that contains Clionaida^57^. Rapid and unique mitochondrial evolution has also been observed in other sponges, such as in Calcaronea with linear chromosomes^58,59^, and in Hexactinellida with a frameshifting translation strategy to cope with their deep-water environments^60^. Furthermore, phylogeny reconstructions of Clionaidae have found *C. varians* to be more closely related to *Spheciospongia* than *C. patera* (Fig. 2), though all are nested within Clionaidae. These patterns support the identical gene arrangement between *C. varians* and *S. vesparium*, but also suggest that the rearrangement occurred recently. Nevertheless, this is only the second complete mitochondrial genome sequenced for *Cliona* and third for Clionaidae to date. More representative data for Clionaida are needed to determine if there is indeed rapid mitochondrial gene evolution within the order (Fig. 3).

The lack of variability for the COI gene among sponge species concurs with numerous past studies, as sponges have relatively slow rates of mitochondrial evolution (Fig. 4)^61–63^, which could be caused by their comparatively long generation times and low metabolic rates^64^. Nevertheless, in contrast to other members of Clionaidae, the *C. patera* population in Singapore had virtually no variation for the three gene loci analysed (Fig. 4, Tables S1–S3). For example, León-Pech et al*.*^65^ found a minimum of 0.2% divergence within *C. vermifera*, and we detected at least some sequence variability in all species compared except *C. yorkin*. Alternative markers commonly used in phylogenetic and taxonomic studies of sponges include nuclear markers such as ITS and 28S loci of the ribosomal RNA (rRNA) region^66^, with the former being common in phylogeographic studies due to their higher degree of intraspecific variability^65,67^ (but see Wörheide et al.^45^).

The lack of variability across the whole mitochondrial genome and ribosomal genes examined here could be caused by common maternal inheritance within *C. patera*. Considering that this species was once thought to be extinct, the population bottleneck in the early twentieth century and the resultant small remnant population that arose from a few ancestral individuals would have diminished the gene pool markedly. Low genetic diversity increases the risk of extinction by reducing the capacity of populations to adapt to environmental changes due to the lack of variation. Furthermore, high levels of urbanisation (e.g., land reclamation) and associated anthropogenic pressures (e.g., pollution, high sedimentation rates, ship groundings) along the coastlines of the city state exert further duress on the *C. patera* population in Singapore. These threats are known to be detrimental for marine invertebrates^68,69^, even leading to the local extirpation of several species^8,70^. The relocation of the sponges in Singapore to a marine park would partially ameliorate some of the threats faced by the *C. patera* population, but this measure (i.e., the no-fishing zone) may introduce other stressors such as increased abundance of large spongivores.

With advanced sequencing technologies, it is now possible to trace population genetic patterns from museum samples collected centuries ago, based on remnant ancient DNA (aDNA) using highly rigorous techniques^71^. For sponges, mini-barcodes have been developed for sponge identification of museum specimens^72,73^ that can be extended in future studies to multi-marker assays for reconstructing population histories. Considering the large collections of *C. patera* available in museums around the world due to the wanton harvesting in the nineteenth century, population genetic studies of *C. patera* specimens around the world can be performed to detect bottlenecks in the past^74^. This would shed more light on the detrimental effects that anthropogenic activities have on sponge populations^75,76^ and potentially drive conservation efforts to limit overharvesting globally.

Predation by spongivores, especially larger taxa, are able to shape sponge communities^77–79^. A recent review showed that fishes are known to influence sponge distributions in the Atlantic, with some 50 sponge genera serving as a prey^80^. Furthermore, large predators such as the hawksbill turtle appear to have a preference for certain sponge prey, such as those with a lower spicule content^81^. More specifically, predators of clionaids include both vertebrates (e.g., parrotfish, pufferfish, damselfish and turtles) and invertebrates (e.g., gastropods, decapods, isopods and echinoids)^82–86^. For example, Mortimer et al*.*^87^ investigated sponge consumption based on 18S metabarcoding and found members of Clionaida within the gut content of a number of different spongivorous fishes in Wakatobi Marine National Park, Indonesia. Clionaids appear to be palatable to spongivores as numerous bite marks on *C. patera* have been recorded (Table S4).

Rapid regeneration and anti-predatory mechanisms are critical for the survival of sponges, particularly in large reef sponges such as *Neofibularia nolitangere*, *Ircinia strobilina* and *Agelas clathrodes* in the Caribbean^88^. In *C. celata*, rapid regeneration enabled papillae consumed to regenerate as quickly as within 12 days^89^. Similarly, we found that *C. patera* generally had remarkably high rates of recovery, taking only about three weeks to seal a 20 mm diameter core hole (Fig. 5C,D). In *C. celata*, regeneration rates were generally correlated with high current flow (present in SIMP), possibly aiding regeneration by reducing the amount of energy expended for feeding or waste removal^89^. *Cliona patera* could employ a similar strategy to their Caribbean counterparts, coupling mechanical defences (spicules) with rapid regeneration to maximise their survival chances under predatory stress.

Upon relocation, three of the six sponges were able to either maintain constant height, and one even grew by 8% (Fig. 5A). Nevertheless, two individuals fared poorly, with their cups almost entirely consumed (e.g. Fig. 5B). A recent paper by Wulff^79^ debunked the popular binary of sponges being either “palatable” or “deterrent” based on frequency of consumption of sponge pellets, proposing that sponge defenses are predator- and habitat-specific. By transplanting the sponges to SIMP, the sponges might have inadvertently been exposed to increased opportunistic predation, particularly by aggregating them together. In addition, wounding of the sponge during regeneration experiments could have altered their gene expression, requiring allocation of metabolic resources toward regeneration and possibly triggering the release of other spongivore-attracting metabolites^90,91^. Future conservation efforts on this sponge need to carefully consider the diversity of potential spongivores in the target site prior to any relocation of individuals. We note that only two out of the six individuals at SIMP were preyed on severely, so more data on the effects of sponge aggregation on predation are needed to further assess this strategy. Finally, considering the trade-off in resource allocation between growth and gametogenesis in clionaids^92^, it is highly likely that individuals E and F (Fig. 5A,B) would prioritise recovery and growth and not be sexually active for some time.

The reproduction biology of *Cliona* has been studied extensively for several species, including *Cliothosa delitrix*^93^, *C. tenuis*^92^, *C. vermifera*^94^, *C. celata* and *C. viridis*^95^. In these studies, *C. vermifera* and *C. tenuis* were determined to be gonochoric, *C. celata* and *C. viridis* hermaphoroditic, and *Cliothosa delitrix* mostly gonochoric with some hermaphrodites observed. With the exception of *C. vermifera*, all other clionaids were oviparous. In addition, gamete release generally occurred under warmer temperatures, with up to 90% of sexual reproduction occurring annually. Interestingly, despite the high percentage of *Cliothosa delitrix* individuals found to contain reproductive structures^93^, a population study across the Greater Caribbean found > 12% of the samples were clones (n = 495)^96^, contrary to an earlier study which found no clones (n = 47)^97^. Based on available literature, we propose that *C. patera* is unlikely to reproduce by fragmentation, similar to other stalked sponges due to its inability to regenerate the attachment stalk and poor attachment^98,99^. Therefore, it is critical for cross-fertilization to be the main driver for increasing not only the threatened species’ genetic diversity but also population abundance to ensure its long-term viability.

The low abundance and genetic diversity of this sponge population are pressing concerns for the species. Despite stringent legislative protection from associated anthropogenic impacts (e.g., seabed dredging, anchoring, coastal reclamation and harvesting) on the transplanted individuals, inbreeding between the small pool of individuals could continue to erode their genetic fitness, resulting in increased risk of extinction. In the future, by enacting partnerships between countries harbouring *C. patera* populations, with in situ individuals currently found only in Cambodia, Singapore and Thailand, the global genetic diversity of this species can be estimated across populations. Using high-throughput sequencing techniques such as those applied in this study, the global population genomics of *C. patera* can be examined to guide conservation action plans and enhance the species’ genetic diversity. Critically, its exceptional rarity throughout the region may require ex situ conservation efforts, including transplanting and propagating genetically distinct individuals in aquarium settings. Public aquaria harbour a rich diversity of marine organisms, often including threatened species, and many have been involved in conservation and reintroduction programmes^100^. However, while some clionaids have been kept in laboratory aquaria successfully for experiments, these are mostly performed for encrusting forms^101–103^. Clearly, the reproductive biology of *C. patera* and its viability in the aquarium must be carefully assessed to mitigate the risk of losing the entire population. If successful, individuals reared in captivity may be relocated back to the wild, within suitably identified sites after careful assessment as outlined here.

## Supplementary Information

Supplementary Information.

## Data Availability

The datasets generated and/or analysed during the current study are available in SRA/GenBank under BioProject PRJNA796519 (raw reads), accession numbers OM273296–OM273301 (mitochondrial genomes), ON705641–ON705646 (28S rRNA) and OP937170–OP937175 (ITS sequences).
